# Efficacy and safety of indacaterol/glycopyrronium fixed-dose combination in mild-to-moderate COPD patients symptomatic on tiotropium in Korea: study protocol for a randomized controlled trial

**DOI:** 10.1186/s13063-017-1800-3

**Published:** 2017-02-22

**Authors:** Chin Kook Rhee, Hye Yun Park, Jeong-Woong Park, Ji-Hyun Lee, Tae-Hyung Kim, Sei Won Lee, Ji Ye Jung, Song Kim, Yong Il Hwang, Ki-Suck Jung

**Affiliations:** 1 Division of Pulmonary, Allergy and Critical Care Medicine, Department of Internal Medicine, Seoul St. Mary’s Hospital, College of Medicine, The Catholic University of Korea, Seoul, South Korea; 2 Division of Pulmonary and Critical Care Medicine, Department of Medicine, Samsung Medical Center, Sungkyunkwan University School of Medicine, Seoul, South Korea; 3 Division of Pulmonary and Allergy Medicine, Gachon University, Gil Medical Center, Incheon, South Korea; 4 Department of Internal Medicine, Bundang CHA Medical Center, CHA University College of Medicine, Seongnam, South Korea; 5 Department of Internal Medicine, Hanyang University College of Medicine, Seoul, South Korea; 6 Department of Pulmonary and Critical Care Medicine, Asan Medical Center, University of Ulsan College of Medicine, Seoul, South Korea; 7 Division of Pulmonology, Department of Internal Medicine, Severance Hospital, Yonsei University College of Medicine, Seoul, South Korea; 8 Novartis Korea Ltd., Seoul, South Korea; 9 Division of Pulmonary, Allergy and Critical Care Medicine, Department of Medicine, Hallym University Sacred Heart Hospital, Hallym University Medical School, 896 Pyeongan-dong, Dongan-gu, 431-070 Anyang-si, Gyeonggi-do South Korea

**Keywords:** Chronic obstructive pulmonary disease, LABA/LAMA combination, Bronchodilator agents, Korea, Indacaterol/glycopyrronium, Tiotropium

## Abstract

**Background:**

Long-acting bronchodilator monotherapy (long-acting β_2_-agonist [LABA] or long-acting muscarinic antagonist [LAMA]) is extensively used for treatment of patients with chronic obstructive pulmonary disease (COPD) with mild-to-moderate airflow limitation. However, a substantial number of patients remain symptomatic despite treatment with a single bronchodilator, necessitating a change in therapy.

**Methods:**

This 12-week, randomized, multicenter, open-label, phase IV study aims to show that the once-daily indacaterol/glycopyrronium (IND/GLY) 110/50 μg fixed-dose LABA/LAMA combination results in an improved lung function in symptomatic patients with mild-to-moderate COPD who switch from once-daily tiotropium 18 μg. The study aims to enroll a total of 404 symptomatic patients in Korea with mild-to-moderate COPD who received tiotropium for at least 12 weeks prior to the study initiation. The primary objective of this study is to demonstrate the superiority of IND/GLY over tiotropium in terms of trough forced expiratory volume in 1 second (FEV_1_) following 12 weeks of treatment. Secondary endpoints include the pre-dose trough FEV_1_ after 4 weeks of treatment, transition dyspnea index (TDI) total score, COPD assessment test (CAT) total score, and rescue medication use following the 12-week treatment, and safety assessment over the 12-week treatment.

**Discussion:**

This study intends to establish the use of LABA/LAMA combination therapy in symptomatic patients with mild-to-moderate COPD by demonstrating the superiority of IND/GLY over tiotropium monotherapy.

**Trial registration:**

ClinicalTrials.gov, NCT02566031. Registered on 10 August 2015.

**Electronic supplementary material:**

The online version of this article (doi:10.1186/s13063-017-1800-3) contains supplementary material, which is available to authorized users.

## Background

Chronic obstructive pulmonary disease (COPD) is a progressive respiratory disorder characterized by persistent airflow limitation. COPD is a highly prevalent disorder affecting around 210 million adults worldwide and accounting for nearly 3 million deaths per year [1, 2]. Among Korean adults aged ≥40 years, COPD prevalence in 2008, determined using spirometry was 13.4% [3]. In the Korean population, according to the Korea National Health and Nutrition Examination Survey, this corresponds to an estimated prevalence of 3.2 million cases [3].

Current Global Strategy for the Diagnosis, Management and Prevention of COPD (GOLD) strategy, defines severity of COPD in patients based on the assessment of lung function, symptoms, and exacerbation risk and classifies patients into four groups, A, B, C, or D [4]. Patients in groups A and C have fewer symptoms, and those in groups B and D have more symptoms. Patients with COPD, particularly with mild-to-moderate airflow limitation (trough forced expiratory volume in 1 second [FEV_1_] ≥ 50% of the predicted value), with <2 exacerbations in the previous year not requiring hospitalization for exacerbation, and with COPD assessment test (CAT) score ≥10 or modified Medical Research Council (mMRC) grade ≥2 are classified in GOLD group B, i.e., patients with low risk and more symptoms [4, 5].

GOLD strategy recommends the use of long-acting mono-bronchodilators, including a long-acting β_2_-agonist (LABA) or a long-acting muscarinic antagonist (LAMA), as the first-line therapy for the treatment of GOLD group B patients [6]. However, a significant proportion of patients receiving long-acting bronchodilator monotherapy continue to experience symptoms and poor quality of life [7]. Hence, the over-prescription of inhaled corticosteroids (ICS) (in combination with a LABA or a LAMA) has been frequently reported in group B patients, leading to increased risk of pneumonia and other side effects [8]. Recently, combination of bronchodilators such as a LABA and a LAMA that target different bronchodilation pathways has been shown to enhance bronchodilation in symptomatic patients [9]. The evidence from prospective clinical studies also indicates greater improvement in lung function with LABA/LAMA combination therapy compared with increasing the dose of a single bronchodilator in patients with moderate-to-severe COPD [10].

The once-daily fixed-dose combination of indacaterol (IND, a LABA)/glycopyrronium (GLY, a LAMA) 110/50 μg combines these two bronchodilators in a single inhaler and is approved for maintenance treatment of patients with moderate-to-severe COPD. IND/GLY was first marketed in Europe in 2013 and has since been marketed in more than 90 countries globally, including in Korea since May 2014.

### Rationale

Treatment with a single bronchodilator such as a LAMA is recommended for group B patients by the GOLD strategy. This study aims to evaluate the effect of the alternative treatment with a LABA/LAMA combination (IND/GLY 110/50 μg) in GOLD group B (FEV_1_ ≥ 50% of the predicted normal value and CAT ≥10) patients who remain symptomatic despite maintenance treatment with a LAMA. As tiotropium is currently the most widely used LAMA in COPD treatment in Korea, this has been selected as the comparator in this study.

This study aims to provide evidence in addition to the existing data to demonstrate the superiority of once-daily IND/GLY 110/50 μg over once-daily tiotropium18 μg in improving lung function over once-daily tiotropium18 μg in symptomatic patients with mild-to-moderate COPD. Furthermore, this study aims to explore the effects of IND/GLY versus tiotropium on symptom burden, breathlessness, and use of rescue medication in these patients.

## Methods

### Study design

**Fig. 1:**
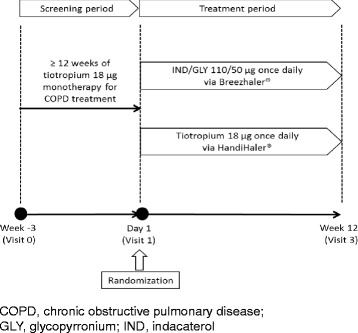
Study design

**Fig. 2:**
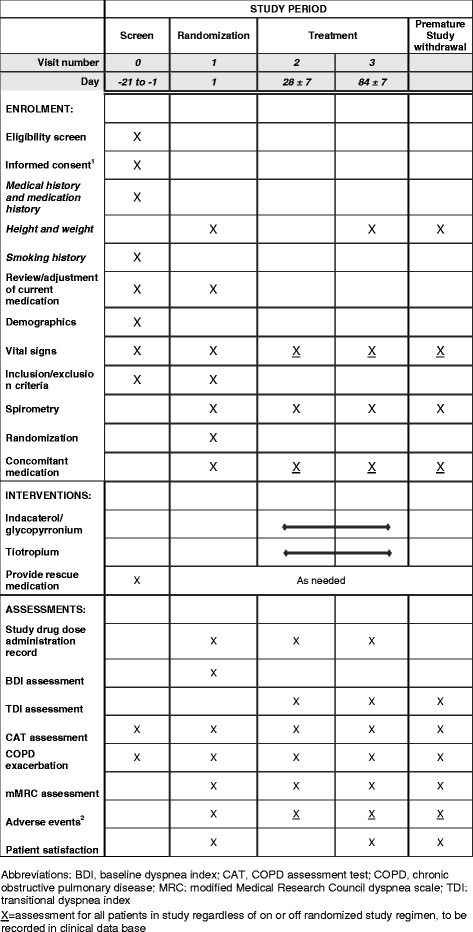
Schedule of enrolment, interventions and assessments

This is a 12-week, randomized, multicenter, open-label, parallel-group, phase IV study. At an initial pre-screening visit (visit 0), a written informed consent will be obtained, followed by assessing the inclusion/exclusion criteria and baseline COPD medication and demographics data. Following a 3-week screening period, at visit 1, eligible patients will be randomized in a 1:1 ratio to receive either once-daily IND/GLY 110/50 μg delivered via the Breezhaler^®^ device (Novartis ﻿Pharma AG, Basel, Switzerland) or once-daily tiotropium 18 μg delivered via the HandiHaler^®^ device (Boehringer Ingelheim, Ingelheim, Germany) during a 12-week treatment period (Fig. 1). Spirometry measurements assessing the post-bronchodilator FEV_1,_ along with the baseline dyspnea index (BDI), mMRC and CAT assessments will also be performed at visit 1.During the study, patients will be permitted to use salbutamol/albuterol (short-acting bronchodilator) as a rescue medication, as needed. The schedule of visits and measurements is given in Fig. 2.

### Ethical conduct of the study and informed consent

This study will be conducted in 20 centers, comprising of general hospitals. The study will be conducted in accordance with the Declaration of Helsinki and Good Clinical Practice guidelines and has been approved by the institutional review boards and ethics committees at participating centers (list included in Additional file 1).

Written informed consent will be obtained from every participant in the study.

### Patients

The first patient’s first visit for the study took place in January 2016 and the recruitment is expected to conclude by the end of 2017. In line with the inclusion/exclusion criteria, this study aims to enroll a total of 404 symptomatic patients with mild-to-moderate COPD in Korea who would have received tiotropium for at least 12 weeks prior to the study initiation and would be given either IND/GLY or tiotropium for 12 weeks.

#### Inclusion criteria

Male or female patients to be enrolled in the study should meet the following inclusion criteria: age ≥40 years; confirmed diagnosis of COPD with mild or moderate airflow limitation, post-bronchodilator FEV_1_ ≥ 50% of the predicted value, and FEV_1_/forced vital capacity (FVC) ratio <0.7; CAT score ≥10 at screening along with a history of <2 exacerbations; and no hospitalization for exacerbation in the previous 12 months. Patients must be current or ex-smokers with a smoking history of ≥10 pack-years and must be on tiotropium monotherapy for at least 12 weeks before the study initiation.

#### Exclusion criteria

Key exclusion criteria include a history of treatment with any ICS in 12 weeks prior to the study initiation; a history or current diagnosis of cardiac arrhythmias; or a history of familial long QT syndrome or type I or uncontrolled type II diabetes or narrow-angle glaucoma. Patients requiring long-term oxygen therapy prescribed for >12 hours per day or experiencing COPD exacerbation during the 12-week screening period that requires hospitalization or with a history of respiratory tract infection within 4 weeks prior to the screening or who develop a respiratory tract infection during the screening or with a history of any concomitant pulmonary disease or with a history or current diagnosis of asthma will also be excluded.

#### Randomization

All eligible patients will be randomized (1:1) using the interactive randomized technology (IRT) to one of the treatment arms. The IRT will assign a randomization number to the patient, which will be used to link the patient to a treatment arm and will specify a unique medication number for the first package of study drug to be dispensed to the patient.

To ensure that the treatment assignment is unbiased and concealed from both patients and investigators the patient randomization list will be produced by the IRT provider using a validated system that automates the random assignment of patient numbers to randomization numbers. These randomization numbers are linked to the different treatment arms, which in turn are linked to medication numbers.

### Objectives

The primary objective of the study is to demonstrate the superiority of IND/GLY over tiotropium monotherapy in terms of trough FEV_1_ (defined as the mean of the pre-dose 45 and 15 minutes FEV_1_ values), following 12 weeks of treatment in patients with mild-to-moderate symptomatic COPD. Secondary objectives are to evaluate pre-dose trough FEV_1_ after 4 weeks of treatment, transition dyspnea index (TDI) total score, total CAT score, and rescue medication use following the 12-week treatment. Safety and tolerability will also be assessed over the 12-week treatment. Safety assessment will include monitoring of all the adverse events (AEs) and serious adverse events (SAEs) (regardless of causality); measuring vital signs, including pulse rate and blood pressure; and performing complete physical examinations, clinical laboratory tests, and electrocardiograms. The occurrence of AEs will be sought by non-directive questioning of the patient at each visit during the study or may also be detected by patient volunteering during or between visits or through physical examination, laboratory test, or other assessments.

Exploratory objectives will assess patient preference and satisfaction with IND/GLY in the group of patients receiving IND/GLY, after the patients have been treated with both regimens. Other exploratory outcome measures will comprise of mMRC dyspnea scale scores after 4 and 12 weeks of the treatment and the correlation between patients’ mMRC and CAT scores.

### Statistical analysis

The efficacy analysis will be performed on the full analysis set (FAS), whereas the per protocol set (PPS) will be used for supportive analysis of the primary variable. The FAS will comprise all randomized patients who receive at least one dose of study treatment and will have at least one evaluable post-baseline assessment. The PPS will include all patients in the FAS without any major protocol deviations. All safety analyses will be performed on the safety set comprising of all patients who receive at least one dose of the study treatment.

An analysis of covariance (ANCOVA) model will be used for the primary analysis with treatment, smoking status as fixed effects, baseline trough FEV_1_ as a covariate and center as a random effect. The superiority of IND/GLY to tiotropium will be demonstrated if the *p* value is less than 0.05, and the 95% confidence interval lies entirely to the right of (higher than) 0 mL. In addition, the primary analysis will be repeated for the PPS as a supportive analysis. The missing FEV_1_ values will be imputed by the last observation carried forward (LOCF) method.

Moreover, trough FEV_1_ at week 4 will be analyzed using an ANCOVA model similar to the primary analysis, with visit and treatment by visit interactions as additional fixed effects. The TDI total score at week 12 will be analyzed using the same ANCOVA model with the baseline dyspnea index (BDI) focal score as the baseline covariate. The proportion of patients with a clinically important improvement of at least 1 in the TDI focal score will be analyzed using logistic regression. The CAT score after 12 weeks of treatment will be summarized based on treatment and analyzed using similar ANCOVA model, with the baseline covariate replaced by baseline CAT score.

### Sample size calculation

This study aims to randomize 182 patients in each treatment arm (IND/GLY and tiotropium) to demonstrate the superiority of IND/GLY over tiotropium in terms of pre-dose trough FEV_1_ following 12 weeks of treatment at 5% level of significance with 80% power on a two-sided test. This is based on the assumptions that the estimated treatment difference between IND/GLY and tiotropium is 80 mL and corresponding standard deviation is 271 mL [10]. Therefore, considering a dropout rate of 11%, approximately 404 patients are to be enrolled in the study.

## Discussion

Once-daily fixed-dose combination IND/GLY 110/50 μg has been shown to significantly improve lung function and patient-reported outcomes (including dyspnea and health status) versus placebo [11, 12]. In addition, IND/GLY showed significant improvements in bronchodilation when compared with its monocomponents and tiotropium in moderate-to-severe COPD patients [11, 13]. These improvements were rapid in onset and sustained throughout the 26-week SHINE study [11] and the 64-week SPARK study [13]. IND/GLY also significantly improved TDI total scores compared with tiotropium [11, 12]. IND/GLY demonstrated a favorable safety and tolerability profile versus placebo in patients with moderate-to-severe COPD over the 52-week ENLIGHTEN study conducted in various countries, including Korea [14].

To add to this existing evidence presenting the efficacy of IND/GLY compared with tiotropium, this randomized, multicenter, open-label, phase IV study intends to demonstrate the efficacy of the switch to IND/GLY from maintenance tiotropium therapy in symptomatic patients with mild-to-moderate COPD. This phase IV study design would directly switch the patients receiving tiotropium to IND/GLY without any washout period and thus differs from the design of phase III clinical studies, in which, a switch to the new therapy requires a washout period. This moves the comparison of IND/GLY to the standard-of-care tiotropium closer to real-life clinical practice.

GOLD strategy categorizes patients with COPD under either A, B, C, or D category based on disease severity, health status, and risk of exacerbations; however, few clinical trials utilize these parameters for selection of appropriate patients. Furthermore, although the combination of a LABA and a LAMA is recommended for treatment of GOLD group B patients, a significant number of such patients continue to experience symptoms and breathlessness. The key feature of this study is to emphasize enrollment of only GOLD group B patients and to specifically assess the efficacy of IND/GLY in this subset of the patients. Going beyond, this study would allow demonstrating clear evidence on the benefits of IND/GLY over tiotropium monotherapy in these patients.

In our opinion, the open-label design would not have any effect on the outcomes because the primary measure involves assessment of trough FEV_1_, which is inherent to the treatment and not influenced by the investigator and participant perception. Further, this open-label design also enables to assess patient preference and satisfaction in patients receiving IND/GLY, which is one of the exploratory objectives of the study. This study is well designed to demonstrate the use of LABA/LAMA combination and sufficiently powered to confirm the superiority of IND/GLY over tiotropium in COPD patients with mild-to-moderate airflow limitation. This study plans to answer the question whether the use of a LABA/LAMA combination would be preferable over continuing long-acting bronchodilator monotherapy in symptomatic COPD patients.

### Trial status

The first patient’s first visit for the study took place in January 2016 and the recruitment is expected to conclude by the end of 2017

## Additional files

Additional file 1: List of participating centers in Korea. (DOC 63 kb)
Additional file 2: SPIRIT 2013 checklist: recommended items to address in a clinical trial protocol and related documents. (DOC 120 kb)

## Abbreviations

AEs: Adverse events
ANCOVA: Analysis of covariance
BDI: Baseline dyspnea index
CAT: COPD assessment test
COPD: Chronic obstructive pulmonary disease
FAS: Full analysis set
FEV_1_: Forced expiratory volume in 1 second
FVC: Forced vital capacity
GOLD: Global Strategy for the Diagnosis, Management and Prevention of COPD
ICS: Inhaled corticosteroid
IND/GLY: Indacaterol/glycopyrronium
IRT: Interactive randomized technology
LABA: Long-acting β_2_-agonist
LAMA: Long-acting muscarinic antagonist
LOCF: Last observation carried forward
mMRC: Modified Medical Research Council
PPS: Per protocol set
SAEs: Serious adverse events
TDI: Transition dyspnea index

## References

[1] World Health Organization (2015). Chronic obstructive pulmonary disease (COPD) Fact sheet No. 315.

[2] López-Campos JL, Tan W, Soriano JB (2016). Global burden of COPD. Respirology.

[3] Yoo KH, Kim YS, Sheen SS, Park JH, Hwang YI, Kim SH (2011). Prevalence of chronic obstructive pulmonary disease in Korea: the fourth Korean National Health and Nutrition Examination Survey, 2008. Respirology.

[4] Global Initiative for Chronic Obstructive Disease (2016). Global strategy for the diagnosis, management, and prevention of chronic obstructive pulmonary disease.

[5] Bellinger CR, Peters SP (2015). Outpatient chronic obstructive pulmonary disease management: going for the GOLD. J Allergy Clin Immunol Pract.

[6] National Institute for Health and Care Excellence (2010). Chronic obstructive pulmonary disease: management of chronic obstructive pulmonary disease in adults in primary and secondary care.

[7] Dransfield MT, Bailey W, Crater G, Emmett A, O’Dell DM, Yawn B (2011). Disease severity and symptoms among patients receiving monotherapy for COPD. Prim Care Respir J.

[8] Wei Y, Kuo P, Tsai Y (2015). Factors associated with the prescription of inhaled corticosteroids in GOLD group A and B patients with COPD – subgroup analysis of the Taiwan obstructive lung disease cohort. Int J Chron Obstruct Pulmon Dis.

[9] Bjerg A, Lundback B, Lotvall J (2012). The future of combining inhaled drugs for COPD. Curr Opin Pharmacol.

[10] Cazzola M, Molimard M (2010). The scientific rationale for combining long-acting beta2-agonists and muscarinic antagonists in COPD. Pulm Pharmacol Ther.

[11] Bateman ED, Ferguson GT, Barnes N, Gallagher N, Green Y, Henley M (2013). Dual bronchodilation with QVA149 versus single bronchodilator therapy: the SHINE study. Eur Respir J.

[12] Mahler DA, Decramer M, D’Urzo A, Worth H, White T, Alagappan VK (2014). Dual bronchodilation with QVA149 reduces patient-reported dyspnoea in COPD: the BLAZE study. Eur Respir J.

[13] Wedzicha JA, Decramer M, Ficker JH, Niewoehner DE, Sandstrom T, Taylor AF (2013). Analysis of chronic obstructive pulmonary disease exacerbations with the dual bronchodilator QVA149 compared with glycopyrronium and tiotropium (SPARK): a randomised, double-blind, parallel-group study. Lancet Respir Med.

[14] Dahl R, Chapman KR, Rudolf M, Mehta R, Kho P, Alagappan VK (2013). Safety and efficacy of dual bronchodilation with QVA149 in COPD patients: the ENLIGHTEN study. Respir Med.

