# Supplementary material for: Efficacy and safety of indacaterol/glycopyrronium fixed-dose combination in mild-to-moderate COPD patients symptomatic on tiotropium in Korea: study protocol for a randomized controlled trial
Source: Trials. 2017 Feb 22;18:80. doi: 10.1186/s13063-017-1800-3 (PMC5322667; doi:10.1186/s13063-017-1800-3)
Supplement: Additional file 1: — List of participating centers in Korea. (DOC 63 kb) [file 13063_2017_1800_MOESM1_ESM.doc]

# List of Participating Centers in Korea

**Protocol No. : CQVA149AKR01**

**Contact list Version No. / Date: 1 / 03Dec2015**

| **Center no.** | **Center name** | **Investigator** | **Main Contact number** | **IRB name and address** |
| --- | --- | --- | --- | --- |
| 1001 | Hallym University Sacred Heart Hospital | Ki-Suck Jung | 031-380-3717 | IRB of Hallym University Sacred Heart Hospital, 896 Pyeongchon-dong, Dongan-gu, Anyang-si, Gyeonggi-do, South Korea |
| 1002 | NHIS Ilsan Hoispital | Chong Ju Kim | 031-900-0237 | IRB of NHIS Ilsan Hoispital , 100 Ilsan-ro Ilsan-donggu Goyang-si Gyeonggi-do, 410-719 Korea |
| 1003 | Seoul St. Mary's Hospital | Chin Kook Rhee | 02-2258-6067 | IRB of Seoul St. Mary's Hospital, 222, Banpo-daero, Seocho-gu, Seoul, Korea |
| 1004 | Gachon University Gil Medical Center | Jeong Woong Park | 032-460-2634 | IRB of Namdong-daero 774beon-gil, Namdong-gu, Incheon, Korea |
| 1005 | St. Paul's Hospital | Sang Haak Lee | 02-961-4500 | IRB of 180, Wangsan-ro, Dongdaemun-gu, Seoul, Korea |
| 1006 | Yonsei University Wonju Severance Christian Hospital | Suk Joong Yong | 033-741-0927 | IRB of 20, Ilsan-ro, Wonju-si, Gangwon-do, Korea |
| 1007 | Hanyang University Guri Hospital | Tae-hyung Kim | 031-560-2240 | IRB of Gyeongchun-ro 153, Guri-si, Gyeonggi-do Korea, 471-701 |
| 1008 | CHA Bundang Medical Center | Ji-Hyun Lee | 031-780-6140 | IRB of 59, Yatap-ro, Bundang-gu, Seongnam-si, Gyeonggi-do, Korea |
| 1009 | Konkuk University Medical Center | kwang Ha Yoo | 02-2030-7522 | IRB of Neungdong-ro, Hwayang-dong, Gwangjin-gu, Seoul 143-729, Korea |

| **Center no.** | **Center name** | **Investigator** | **Main Contact number** | **IRB name and address** |
| --- | --- | --- | --- | --- |
| 1010 | Chonbuk National University Hospital | Yong Chul Lee | 063-259-1664 | IRB of (634-18, Geumam-dong) 20, Geonjiro Deokjin-gu, Jeonju-si, Jeollabuk-do 561-712, Korea |
| 1011 | Samsung Medical Center | Hye Yun Park | 02-3410-0268 | IRB of 81 Irwon-ro, Gangnam-gu, Seoul, 135-710, Korea |
| 1012 | Severance Hospital, Yonsei University Health System | Ji Ye Jung | 02-2228-1980 | IRB of 50-1, Yonsei-ro, Seodaemun-gu, Seoul, Korea |
| 1013 | Kangbuk Samsung Hospital | Seong Yong Lim | 02-2001-1597 | IRB of 78, Saemunan-gil, Jongno-gu,  Seoul 110-746, Korea |
| 1014 | The Catholic University of Korea Yeouido ST. Mary's Hospital | Hyoung-Kyu Yoon | 02-784-5458 | IRB of Yeouido St. Mary’s Hospital, 62 Yeouido-Dong, Yeongdeungpo-gu, Seoul, Korea |
| 1015 | Gangnam Severance Hospital | Min Gwang Byun | 02-2019-3454 | IRB of 211 Eonju-ro, Gangnam-gu, Seoul South Korea |
| 1016 | Ajou University Hospital | Park Kwang joo | 031-219-5121 | IRB of 164, World cup-ro, Yeongtong-gu, Suwon, Gyeonggi-do, Korea |
| 1017 | Korea University Guro Hospital | Jae Jeong Shim | 02-2626-3029 | IRB of 148, Gurodong-ro, Guro-gu, Seoul, Korea |
| 1018 | SMG - SNU Boramae Medical Center | Deog Kyeom Kim | 02-870-2228 | IRB of (156-707) 20. Boramae-Ro 5-Gil DongJak-Gu. Seoul. Republic of Korea |
| 1019 | Hanyang University Medcal Center | Hojoo Yoon | 02-2290-8349 | IRB of 222-1, Wangsimni-ro, Seongdong-gu, Seoul, Korea |
| 1020 | Ulsan University Hospital | Tae Hoon Lee | 052-250-8633 | IRB of 877, Bangeojinsunhwando-ro, Dong-gu, Ulsan, 44033, Rep. of Korea(44033) |
